# Gearbox Fault Diagnosis Method in Noisy Environments Based on Deep Residual Shrinkage Networks

**DOI:** 10.3390/s24144633

**Published:** 2024-07-17

**Authors:** Jianhui Cao, Jianjie Zhang, Xinze Jiao, Peibo Yu, Baobao Zhang

**Affiliations:** 1 College of Mechanical Engineering, Xinjiang University, Urumqi 830017, China; 107552204151@stu.xju.edu.cn (J.C.); 107552304265@stu.xju.edu.cn (X.J.); 107552204321@stu.xju.edu.cn (P.Y.); 2 College of Software, Xinjiang University, Urumqi 830091, China; 118229@stu.xju.edu.cn

**Keywords:** gearbox fault diagnosis, DRSN-CW model, cross-attention mechanism, frequency domain features, noise

## Abstract

Gearbox fault diagnosis is essential in the maintenance and preventive repair of industrial systems. However, in actual working environments, noise frequently interferes with fault signals, consequently reducing the accuracy of fault diagnosis. To effectively address this issue, this paper incorporates the noise attenuation of the DRSN-CW model. A compound fault detection method for gearboxes, integrated with a cross-attention module, is proposed to enhance fault diagnosis performance in noisy environments. First, frequency domain features are extracted from the public dataset by using the fast Fourier transform (FFT). Furthermore, the cross-attention mechanism model is inserted in the optimal position to improve the extraction and recognition rate of global and local fault features. Finally, noise-related features are filtered through soft thresholds within the network structure to efficiently mitigate noise interference. The experimental results show that, compared to existing network models, the proposed model exhibits superior noise immunity and high-precision fault diagnosis performance.

## 1. Introduction

Gearboxes are fundamental and essential components of mechanical systems, and they are widely utilized in a variety of industrial equipment and transmission systems. Due to their operation under high loads and in complex environments, the reliability of gearbox performance is directly tied to the stability and safety of the entire mechanical system. Gearbox failures can engender system downtime and can lead to serious security incidents and economic losses. Therefore, effective gearbox fault diagnosis is vital in preventing unexpected failures, improving productivity, and significantly reducing maintenance costs, providing substantial engineering applications and measuring economic value [1,2].

As civilization grows, industrial equipment becomes more sophisticated, which makes it more challenging to predict the impacts of uncertainties, time-varying disturbances, and noise in structural and parametric control system [3]. There are many types of AI-based fault detection methods, which can be roughly divided into two categories: traditional machine learning-based methods and deep learning-based methods [4,5,6]. Machine learning methods can extract valuable fault features from large datasets, enabling models to efficiently and accurately identify fault conditions. Numerous scholars have proposed the employment of machine learning in automated fault diagnosis [7,8]. Recent studies have demonstrated that the performance of machine learning methods can be enhanced by preprocessing monitoring data using explicit feature extractors such as time domain analysis and frequency domain analysis [9,10,11,12]. Nevertheless, traditional machine learning approaches rarely have complicated architectures, and they possess inherent limitations. They are operated in relatively closed systems, which results in a lower performance ceiling for the algorithms. A study by Lupea et al. [13] confirms this point, emphasizing that traditional machine learning models often lack the complexity and adaptability required for dynamic and unpredictable environments. Similarly, research by Saucedo-Dorantes et al. [14] shows that traditional machine learning models have simpler architectures and poorer adaptability, leading to a lower performance ceiling. Additionally, Nguyen et al. [15] discuss the limitations of traditional machine learning models, including their inability to handle complex data structures and the need for extensive feature engineering.

Conversely, because of its end-to-end learning capacity, deep learning-based intelligent fault detection is becoming widely recognized. By enabling the automatic extraction of data features during the optimization process, the issues linked to the dependence of traditional machine learning methods on human feature extraction can be avoided. Therefore, the necessity of significant human involvement in model construction is minimized. [16,17,18].

Today, deep learning is extensively applied across various fields. For instance, Cao et al. [19] employed long short-term memory (LSTM) neural networks to extract fault features from wind turbine vibration signals and to classify faults, demonstrating that their method outperforms support vector machines (SVMs). Liu et al. [20] proposed a deep learning method using stacked auto-encoders (SAEs) to solve gearbox fault diagnosis, by extracting significant features directly from frequency domain signals. Shang et al. [21] presented a fault diagnosis approach that utilized recurrent neural networks (RNNs) for rotating machinery, displaying remarkable noise robustness. Wu et al. [22] employed one-dimensional convolutional neural networks (CNNs) to analyze planetary gearbox vibration signals, showing that CNNs achieved higher accuracy in planetary gearbox fault diagnosis than traditional methods.

Sensor-collected signal samples frequently contain noise due to the challenging working environment of the equipment, which impairs neural networks’ ability to extract features during training. Numerous noise-reduction techniques have been set forth for handling this issue. Chen et al. [23] proposed a double-scale wide first-layer convolutional neural network method. By extracting features applicable to low-sampling-rate signals and by combining two types of timescale features, the method improves accuracy and suppresses noise. He et al. [24] proposed a scheme combining FFT and multiple signal classification algorithms to eliminate the impacts of spurious frequency phenomena and to ensure the discovery of accurate fault locations. This approach improves the influence of the source domain by having more vital relevance. Additionally, Zhao et al. [25] proposed a deep learning-based noise data feature learning algorithm, which incorporates a soft threshold to effectively reduce noise interference. Building on this network, Liu et al. [26] achieved the accurate assessment of bearing performance degradation by combining deep residual shrinkage networks and long short-term memory networks. Zhang et al. [27] combined transfer learning, attention mechanisms, and residual networks to enhance training efficiency and recognition accuracy, and the model’s high noise interference during the process of data acquisition was successfully suppressed. The above research shows that combining deep learning models with attention mechanisms and signal analysis is feasible for dealing with noise issues.

However, as the single-channel attention mechanism of the RSBU-CW module can lead to the loss of valuable information, more consideration needs to be given to local and global features. Therefore, this paper proposes a novel and highly accurate compound fault diagnosis method for gearboxes. First, by comparing the time domain and FFT frequency domain inputs, an effective input is selected to reduce the impact of model noise. Then, the model is constructed by combining the cross-attention mechanism and the RSBU-CW module. In terms of tackling noisy data, the signal denoising function of the RSBU-CW module and the cross-channel information interaction of the cross-attention mechanism are combined to ensure that the model maintains high performance. The complementary information from different channels reduces the impact of single-channel noise on the overall detection effect, solves the problem of information loss, and improves diagnostic accuracy. To verify the effectiveness of this method, the proposed method was tested on the SEU gear dataset [28] and the Paderborn University bearing dataset [29] with different levels of Gaussian noise.

The proposed method was compared with the deep residual shrinkage network model combined with long short-term memory (DRSN-LSTM), the deep residual shrinkage network model combined with efficient channel attention (DRSN-ECA), the original DRSN-CW model, and three other network models. The main contributions and advantages of this paper are as follows:

1. This study uses the FFT algorithm to perform frequency domain noise reduction on the signal, which significantly enhances the quality of the fault characteristic signal and improves the model’s noisy-environment fault detection ability.
2. This study introduces a cross-attention mechanism at key nodes before the first residual block and after the last residual block, significantly improving feature capture performance.
3. This study innovatively combines RSBU-CW and cross-attention mechanisms. The proposed CA-DRSN model achieves effective information interaction and feature fusion between different channels through the cross-attention mechanism, and then performs noise reduction and feature enhancement through the RSBU-CW module.
4. Comparative experimental results show that the proposed method exhibits superior diagnostic performance on both datasets and maintains high accuracy even in highly noisy environments.

The organization of the paper is as follows: Section 2 introduces the theory of frequency domain analysis, explains the fundamental principles of cross-attention, and details the structure of the CA-DRSN model. Section 3 details the algorithm flow of the CA-DRSN model. Section 4 uses the proposed method to identify different fault categories in the gearbox and compares the diagnostic results. Section 5 outlines the conclusions of this study and provides an outlook for future work.

## 2. Key Theory and Techniques

### 2.1. Fast Fourier Transform

The fast Fourier transform (FFT) is an engineering implementation of the discrete Fourier transform (DFT) and was designed to simplify the computational process, making it more efficient for practical applications [30]. Gou et al. [31] presented a method that adopted the FFT algorithm and conducted a spectral analysis of three-phase currents. The spectrum following FFT transformation revealed the strength of different frequency components, allowing for the selection of the most critical fault characteristic frequencies as model inputs, thereby improving model efficiency. Zhu and Peng [32] addressed the issue of wind abandonment and transmission interruptions caused by short circuits in the power lines of wind farms when double-fed asynchronous generators were utilized. They employed FFT to extract the fundamental wave amplitude of the zero-sequence current. A feature set was constructed to identify the fault locations more accurately.

The FFT transforms a time domain signal into a frequency domain signal, which reveals the signal’s frequency components and helps distinguish and isolate vibration signals from noise. In the context of digital signal processing, the discrete Fourier transform (DFT) is frequently utilized as the Fourier transform for digital signals. The DFT of a finite-length discrete signal $x_{n}(n=0,1,\ldots ,N-1)$ is defined as follows:

(1) $$\begin{array}{l} X(k)=\sum_{n=0}^{N-1}x(n)W_{N}^{kn}\\ k=0,1,\ldots ,N-1,W_{N}=e^{-j\frac{2\pi }{N}} \end{array}$$

where x(n) is the input signal, X(k) is the output of the DFT, and N is the total number of samples.

The FFT decomposes the sequence x(n) into two parts, the even sequence $x_{2}(n)$ and the odd sequence $x_{1}(n)$, each of length $\frac{Ν}{2}$. Thus, we obtain the following:

(2) $$X\left(k\right)=\sum_{n=0}^{\frac{N}{2}-1}x_{1}\left(n\right)\omega_{N}^{2kn}+\sum_{n=0}^{\frac{N}{2}}x_{2}\left(n\right)\omega_{N}^{\left(2k+1\right)n}$$

Extracting the factorization of Equation (2) yields the following:

(3) $$X\left(k\right)=\sum_{n=0}^{\frac{N}{2}-1}x_{1}\left(n\right)\omega_{N}^{2kn}+\omega_{N}^{kn}\sum_{n=0}^{\frac{N}{2}}x_{2}\left(n\right)\omega_{N}^{2kn}$$

where $\omega_{N}^{2k}=e^{-j\frac{2\pi }{N}2kn}=e^{-j\frac{2\pi }{\frac{N}{2}}kn}=\omega_{N/2}^{kn}$, which is integrated into Equation (3) to obtain the following:

(4) $$\begin{array}{cc} X(k) & =\sum_{n=0}^{\frac{N}{2}-1}x_{1}(n)\omega_{N/2}^{kn}+\omega_{N}^{kn}\sum_{n=0}^{\frac{N}{2}-1}x_{2}(n)\omega_{N/2}^{kn}\\ & =X_{1}(k)+\omega_{N}^{k}X_{2}(k) \end{array}$$

where $X_{1}(k)$ is the result of the odd sequence $X_{1}(n)$ and $X_{2}(k)$ is the result of the even sequence $X_{2}(n)$.

FFT can become a highly efficient tool for analyzing signal frequency components because this decomposition significantly reduces the complexity of the DFT computation.

### 2.2. Attention Mechanism

The cross-attention mechanism [33] is a neural network structure specifically designed to process and analyze sequential data. By capturing the dependencies between locations, this attention mechanism enhances the model’s ability to effectively handle sequential data.

Fast Fourier transform (FFT) is applied in the process of managing the gear data, which include both global and local features. When the time domain signal is transformed into a frequency domain signal, the outcome is represented as a spectrum. Global features, such as total energy and overall shape, represent characteristics within the whole spectrum. Local features include the amplitude of each frequency component in the vibration signal, which reveals frequency components associated with specific faults.

**Figure 1:**
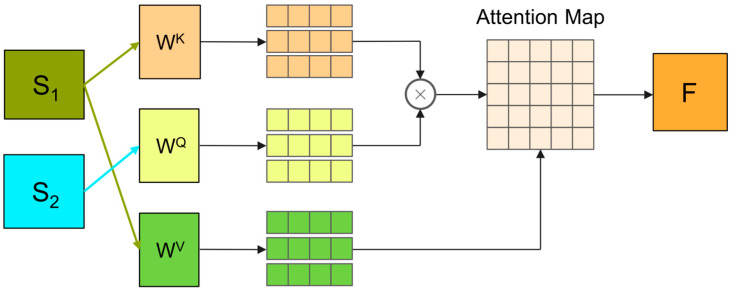
Cross-attention network structure.

Therefore, in practice, global and local features must be considered simultaneously to diagnose faults accurately. By integrating global and local features through cross-attention mechanisms, cross-attention feature associations help a model recognize and learn these features and enhance the model’s ability to distinguish and reduce interference from irrelevant features [34]. More robust and accurate fault diagnosis can be achieved by enabling the model to exploit these advantages. The process of cross-attention feature association is illustrated in Figure 1:

The specific calculation is shown in Equation (5):

(5) $$F=softmax(\frac{(W^{K}S_{1})(W^{Q}S_{2}{)}^{T}}{\sqrt{d}})(W^{V}S_{1})$$

where $S_{1}$ and $S_{2}$ denote global and local features, $W^{K}$, $W^{Q}$, and $W^{V}$ denote learnable parameters, and d denotes feature dimension.

### 2.3. CA-DRSN Model Design

#### 2.3.1. Residual Shrinkage Module with Adaptive Soft Thresholding

The deep residual shrinkage network (DRSN) is a variant of ResNet [25], which integrates a soft thresholding operation into the residual network. This approach enables the residual network to learn an appropriate threshold for each sample based on its channel information, thereby adaptively eliminating noise and redundant information during the feature learning process and improving the overall learning effect and model performance [35]. The functional expression of the soft threshold is given as follows:

(6) $$y=\{\begin{array}{cc} x-\tau & x>\tau \\ 0 & -\tau \leq x\leq \tau \\ x+\tau & x<-\tau \end{array}$$

where x denotes the input feature, and y denotes the output feature; τ is the threshold value, a positive parameter. The derivatives are as follows:

(7) $$\frac{\partial y}{\partial x}=\{\begin{array}{cc} 1, & x>\tau \\ 0, & -\tau \leq x\leq \tau \\ 1, & x<\tau \end{array}$$

The soft threshold function can zero out features within an interval and retain significant negative features, resulting in a better feature map.

**Figure 2:**
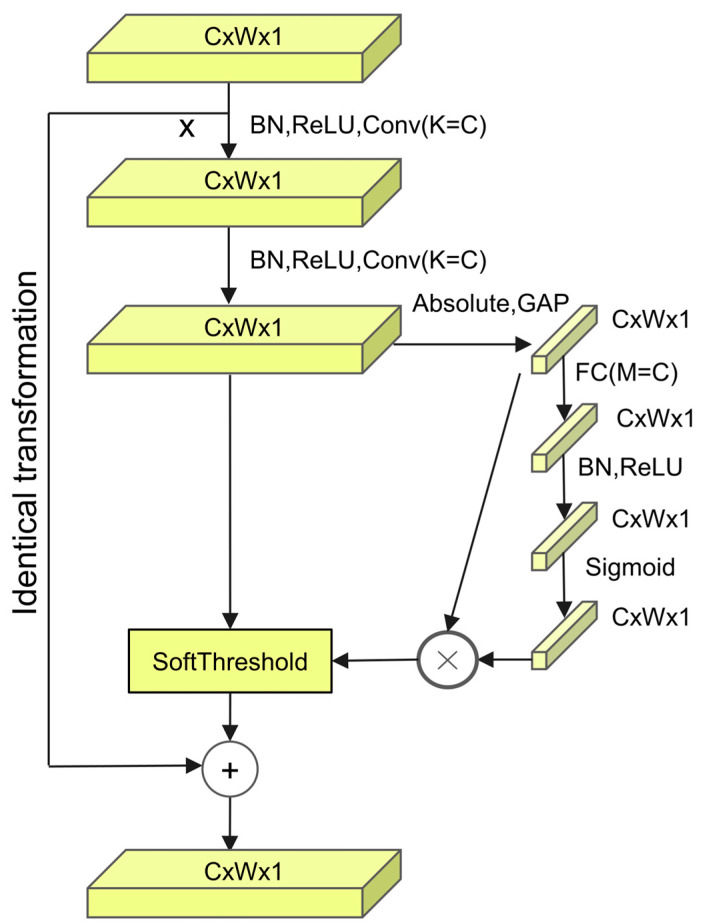
RSBU-CW.

Depending on whether the thresholds are shared between channels, different configurations exist: Residual Shrinkage Building Unit with Channel-Wise Thresholds (RSBU-CW) and Residual Shrinkage Building Unit with Channel-Shared Thresholds (RSBU-CS). RSBU-CW, compared to RSBU-CS, offers greater precision and flexibility and demonstrates superior noise immunity. The specific architecture is shown in Figure 2 below.

In this structure, C, W, and 1 denote the number of channels, width, and height of the feature maps, respectively, while K represents the number of convolution kernels. Initially, the input X undergoes two batch normalization (BN) operations, followed by ReLU activation functions and convolution operations (Conv). K = C indicates that the output channels of the convolution layer are equal to the input channels. This process preserves dimensionality while improving features. Global average pooling (GAP) then compresses the time domain feature vector with absolute value procedures. GAP compresses the features of each channel into a single response value, which is the average of all features in that channel, thereby reducing the model parameters and computational complexity. Next, these compressed feature vectors pass through two fully connected (FC) layers, which explore and learn the associations between channels, effectively extracting attention weights in the channel domain. Subsequently, a Sigmoid function converts the output of the fully connected layers into weight coefficients. These weights are multiplied by the features processed through absolute value and GAP, obtaining the threshold and input into the soft threshold function to eliminate noise and redundant information. The process is described as follows:

(8) $$z=\left|\bar{X}\right|$$

(9) $$a_{c}=\frac{1}{1+e^{-z_{c}}}$$

(10) $$\tau_{c}=z\cdot a_{c}$$

where X represents the output feature map of the second convolution layer, $z_{c}$ denotes the feature of the *c*-th neuron, $a_{c}$ is the scaling parameter of the *c*-th layer, and $\tau_{c}$ is the threshold of the *c*-th channel in the feature map.

#### 2.3.2. Residual Shrinkage Network Model Based on Cross-Attention Module

**Figure 3:**
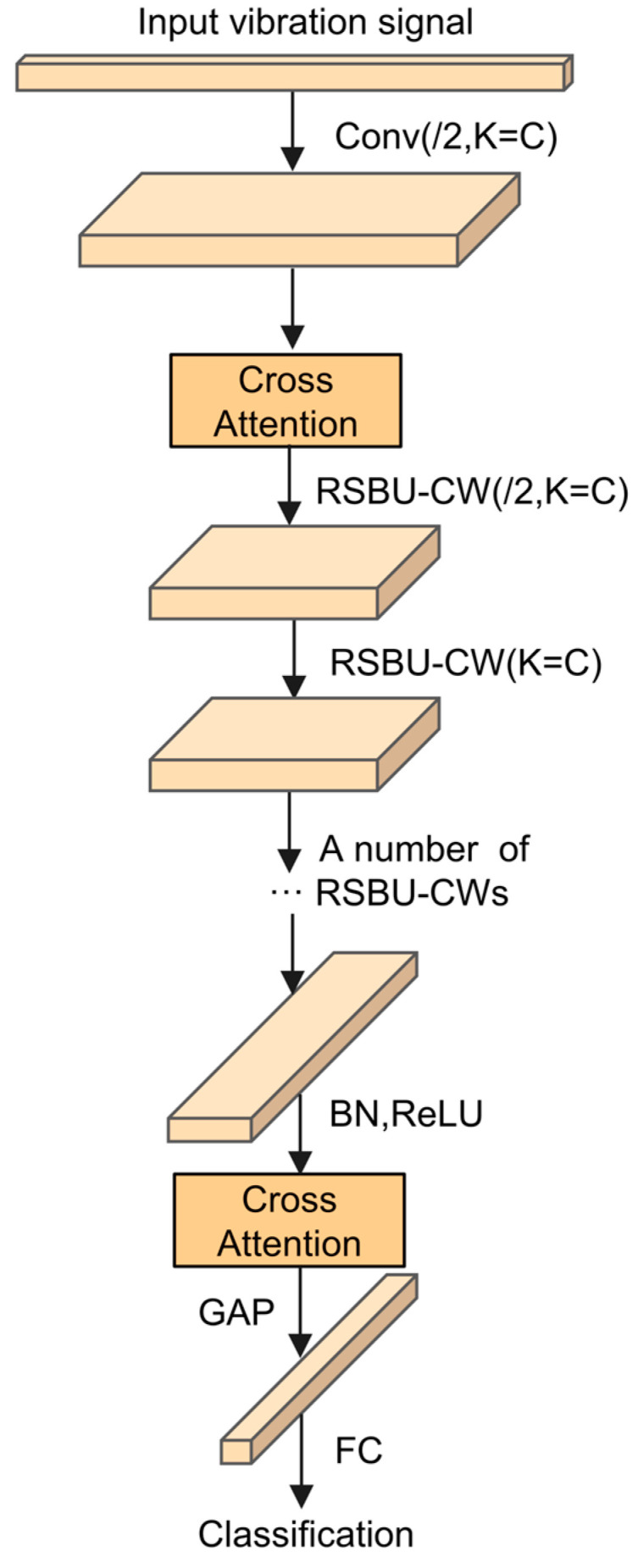
CA-RSBU.

Necessary signals can be overlooked on account of single-channel attention failing to fully utilize the dynamic range between different features. In addition, when dealing with complex or overlapping fault modes, single-channel attention is unable to distinguish subtle differences. Therefore, the cross-attention module is added to this study to produce an enhanced CA-DRSN model. Figure 3 reveals the overall framework of the model.

As shown in Figure 3, the model first extracts the input gear signal from the convolutional layer, processes it with the cross-attention module to capture features, passes it through the residual shrinkage module (RSBU-CW) for noise processing, and then allows it to go through the attention module. Batch normalization (BN), ReLU activation, and global average pooling are then performed. Finally, the prediction result is generated from the fully connected (FC) layer. The introduction of cross-attention into the initial stage of the network helps the model capture key features in the input data, focusing on the most critical parts of the signal from the beginning. Adding an attention module after the final residual block helps to fuse features at different levels, enabling the network to effectively combine global and local information. The accuracy and robustness of fault diagnosis can ultimately be improved.

## 3. Compound Detection Method for Gearboxes Based on CA-DRSN Model

**Figure 4:**
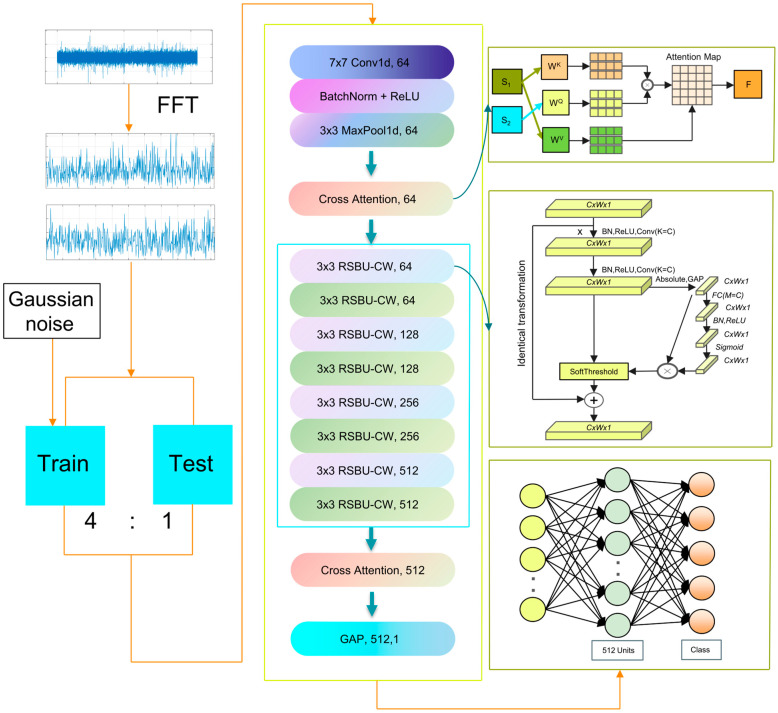
Algorithm flow chart.

In practical applications, the vibration signals obtained from rotating machinery are severely disturbed by noise, making it difficult to accurately extract health status information from these noisy data. This paper proposes a compound detection method for gearboxes based on the CA-DRSN model, which combines fast Fourier transform (FFT) and Gaussian noise to address this issue. Figure 4 shows the model algorithm flowchart with different levels of added Gaussian noise:

First, the time domain signal is transformed into a frequency domain signal because of the use of FFT. The sample data are divided into training and test sets, the ratio of which is 4 to 1. Various levels of Gaussian noise are introduced to the training set, which is subsequently inputted into the CA-DRSN model for feature extraction. The cross-attention module processes the extracted features, allowing the model to focus more precisely on the fault regions in the time-frequency diagram. Finally, the test set is fed into the trained network model to evaluate its accuracy.

## 4. Experimental Results and Discussion

This section first describes in detail the SEU gear dataset used. Then, the fault diagnosis of the CA-DRSN model under the FFT is carried out. The diagnostic results obtained by this method are compared with those of the CA-DRSN model based on the time domain and other existing models. Finally, another gearbox compound fault dataset provided by Paderborn University is used to further verify the effectiveness of the proposed model. Experimental results show that the model has high recognition accuracy and robustness. Additionally, all experiments were run on a computer with an Intel Core i5-12600KF processor, GeForce RTX 3080Ti 12G RAM, and Windows 10 operating system.

### 4.1. Experimental Data Parameters and Settings for the SEU Gear Dataset

**Figure 5:**
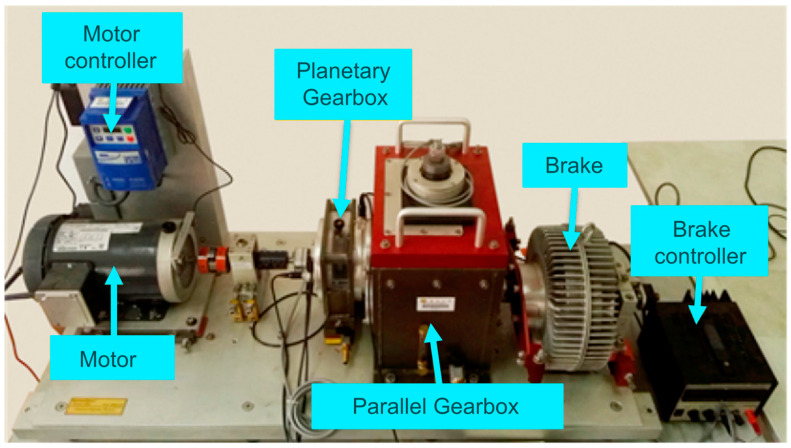
SEU experimental device.

To conduct experiments, in this study, a gearbox dataset provided by Southeast University (SEU) was utilized. The experimental setup, shown in Figure 5, primarily consists of six components: motor, motor controller, planetary gearbox, parallel gearbox, brake, and brake controller.

The gear dataset from SEU was acquired using the Driveline Dynamics Simulator (DDS). The experiments were carried out under two operating conditions: RPM-Load Configuration (RS-LC) setups of 20 Hz-0 V and 30 Hz-2 V, with load setups of 0 V and 2 V. Simultaneously, signals from four types of gear faults and one normal gear condition were collected. Eight rows of vibration signals were recorded, and the second row of vibration signals was implemented. Four types of gear fault data, including chipped, missing, root, and surface faults, were found. A detailed breakdown of the dataset types is presented in Table 1.

For this paper, the SEU gear dataset was preprocessed. The dataset was separated into training and test sets using random segmentation. A sliding window was utilized to truncate the vibration signals without overlap, with each data sample containing 1024 points. Fast Fourier transform (FFT) technology was applied to the dataset, as well. Eighty percent of the samples were randomly selected as the training set, and twenty percent were randomly selected as the test set, with different levels of Gaussian noise added to the training set. Adding different levels of Gaussian noise to the training set simulates the noise in the data collection process. The probability density function of Gaussian noise follows a Gaussian distribution, represented as follows:

(11) $$P(x)=\frac{1}{\sqrt{2\pi }\sigma }exp\left(-\frac{(x-\mu {)}^{2}}{2\sigma^{2}}\right)$$

(12) $$\sigma =\sqrt{\frac{1}{n}\sum_{i=1}^{n}x_{i}^{2}}=\mathrm{Noise}\;\mathrm{level}$$

where x is the random variable, μ is the mean, and σ is the standard deviation.

**Figure 6:**
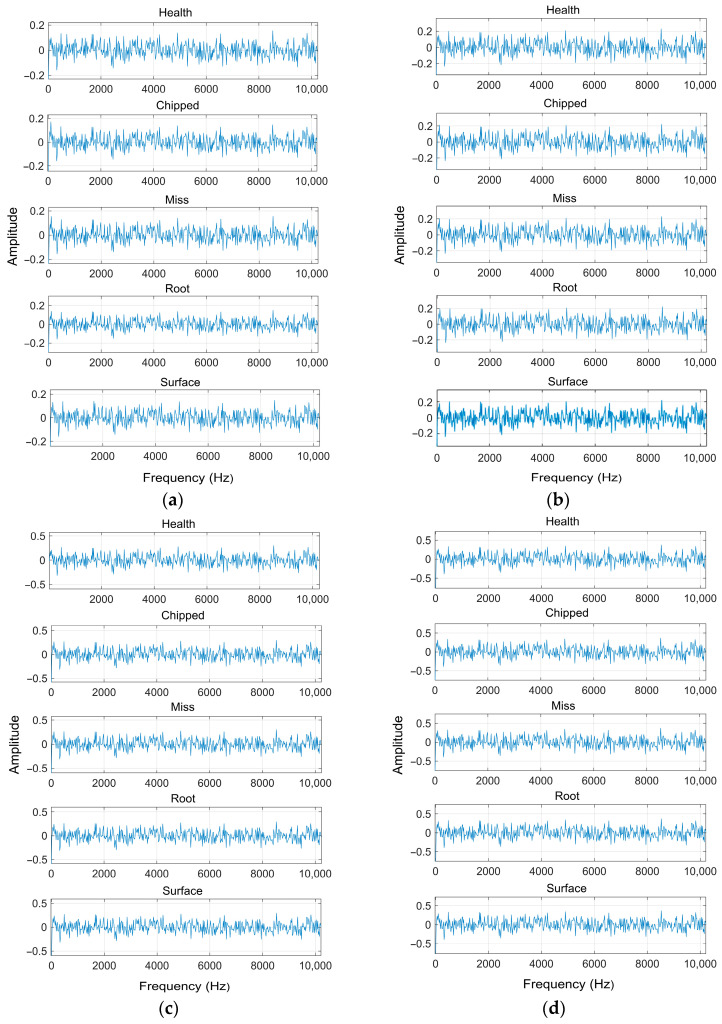
Frequency domain diagrams of the gear vibration signal of different fault types after FFT at different Gaussian noise intensities. (**a**) Frequency domain diagram with noise at 0.1; (**b**) frequency domain diagram with noise at 0.15; (**c**) frequency domain diagram with noise at 0.2; (**d**) frequency domain diagram with noise at 0.25.

In the experiment, the average value of the Gaussian noise was set to 0, and the standard deviation values representing the noise level were set to 0.1, 0.15, 0.2, and 0.25 in order to test the model. Due to the symmetry of the spectrum, only half of the FFT transform is shown, as shown in Figure 6:

The robustness of the model was evaluated by analyzing its sensitivity to noise, which was based on its performance on the validation set after being trained on the training set with added noise.

Table 2 shows how the PyTorch framework was manipulated to construct the model in this paper. Adam was then used as the optimizer. Each experiment’s learning rate, batch size, and discard value were set to 0.001, 32, and 0.15, respectively. Each model was trained for 100 cycles, alternating model training and model testing.

### 4.2. Advantages of the Frequency Domain Analysis Method

**Figure 7:**
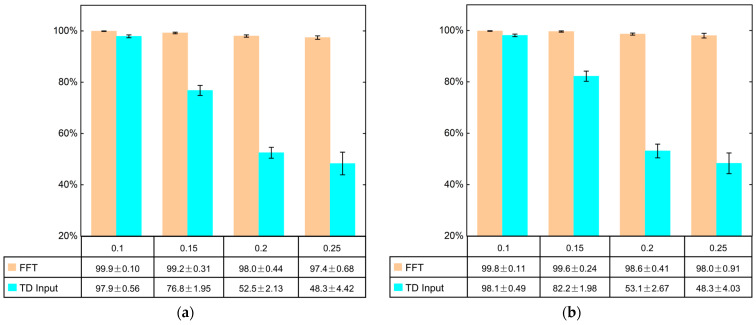
Comparison of FFT and TD input under two working conditions. (**a**) Working condition 1—20 Hz-0 V; (**b**) working condition 2—30 Hz-2 V.

In this paper, the time domain direct input and the frequency domain one-dimensional FFT data comprise the input of the CA-DRSN model. Figure 7 compares the accuracy and standard deviation of the model test set obtained by adding different noise levels in both conditions. In Figure 7, the accuracy of the faults for the frequency domain inputs is consistently above 97%, and the standard deviation is slight, indicating that the results are more stable and reliable. However, the time domain input’s accuracy drops significantly with increasing noise levels. The standard deviation is significant, especially in the case of 0.2 and 0.25, where the accuracy drops to 52.5% and 48.3%, and the standard deviation is ±2.13 and ±4.42, respectively. Also, poor stability is shown.

Therefore, the frequency domain analysis method in this paper is superior to the direct time domain input method in diagnosing one-dimensional gearbox fault signals.

### 4.3. Model Training and Analysis of Results

We conducted a comparative experiment to demonstrate the effectiveness of cross-attention in the fusion model. The CA-DRSN model, which integrates cross-attention, was compared with DRSN-LSTM, DRSN-ECA, the original DRSN-CW model, and three other network models (ResNet18, AlexNet, and BiLSTM).

**Figure 8:**
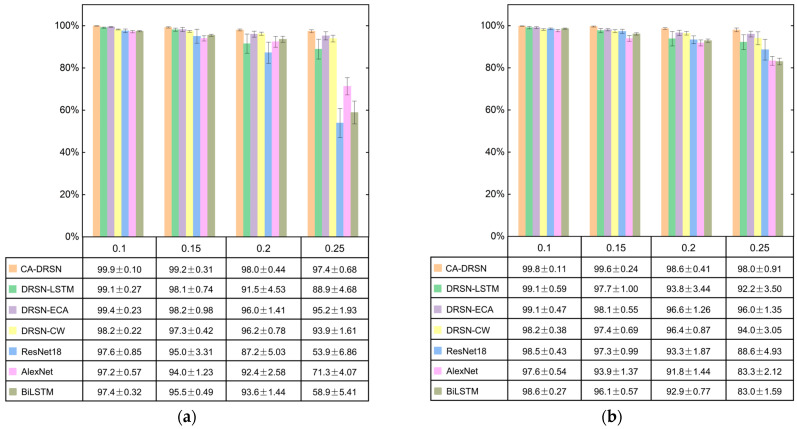
Accuracy and standard deviation of seven model test sets under different working conditions. (**a**) Working condition 1—20 Hz-0 V; (**b**) working condition 2—30 Hz-2 V.

Figure 8 shows the seven models’ diagnostic accuracy and standard deviation at different noise levels under the two operating conditions. CA-DRSN has a significant advantage over the other methods, especially at higher Gaussian noise levels. Under both operating conditions, when the standard deviation (STD) increased from 0.1 to 0.25, the accuracy rates of CA-DRSN were 99.9%, 99.2%, 98.0%, and 97.4%, respectively. Similarly, the accuracy under working condition 2 was 99.8%, 99.6%, 98.6%, and 98.0%, with small standard deviations. This indicates that the inclusion of cross-attention and its appropriate placement significantly enhance the accuracy and robustness of the model.

These experimental results show that the accuracy of other models decreases significantly under high levels of Gaussian noise, and the standard deviation also increases sharply, so their performance in high-noise environments is unstable. In contrast, although the accuracy of CA-DRSN has decreased, it performs better than other models by fewer standard deviation changes and indicates its robustness to noise.

In summary, the CA-DRSN model shows high accuracy and stability under different noise levels. Compared with the other six models, at low noise levels, CA-DRSN performs well; at high noise levels, it remains robust and noise-resistant. These results fully demonstrate the superiority of the CA-DRSN model.

**Figure 9:**
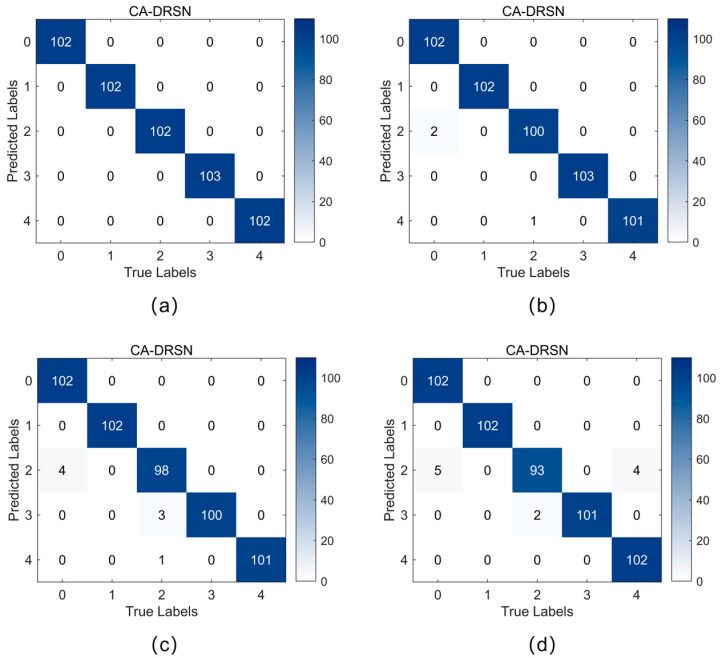
Confusion matrix for fault detection in the CA-DRSN model under working condition 1: (**a**) noise level at 0.1; (**b**) noise level at 0.15; (**c**) noise level at 0.2; (**d**) noise level at 0.25.

**Figure 10:**
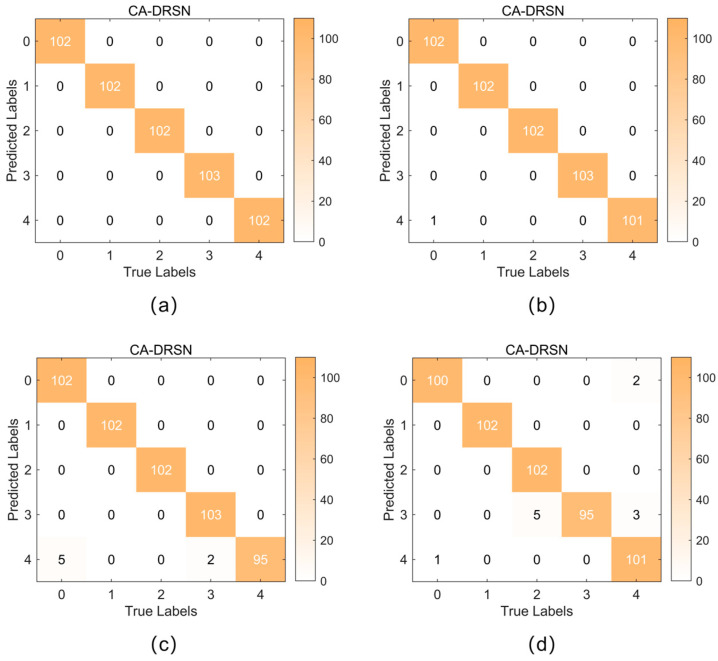
Confusion matrix for fault detection in the CA-DRSN model under working condition 2: (**a**) noise level at 0.1; (**b**) noise level at 0.15; (**c**) noise level at 0.2; (**d**) noise level at 0.25.

Figure 9 and Figure 10 show the confusion matrices of the CA-DRSN model for the two working conditions under different noise levels. The horizontal axis represents the actual fault type, while the vertical axis represents the prediction result: the chipped, health, miss, root, and surface. This paper analyzes the confusion matrices obtained by evaluating the average precision (AP) and average recall (AR) for the five fault types. Precision measures the proportion of correctly predicted samples out of the total predicted samples, while recall measures the proportion of correctly predicted samples out of the total actual samples [36].

As shown in Table 3, at a noise level of 0.1, CA-DRSN achieved an AP and AR of 1.0, which offered the best performance among all models. At a noise level of 0.15, the AP and AR of CA-DRSN slightly decreased but remained above 0.994, maintaining a significant lead under high-noise conditions. In contrast, the AP and AR of DRSN-LSTM and DRSN-ECA remained good at low noise levels but decreased significantly as the noise level increased.

In summary, the CA-DRSN model shows excellent robustness, maintaining high average precision and average recall at different noise levels. In particular, it shows the most minor performance degradation at higher noise levels, which means excellent robustness. Compared with other models, CA-DRSN maintains the leading performance in both low-noise and high-noise environments.

**Figure 11:**
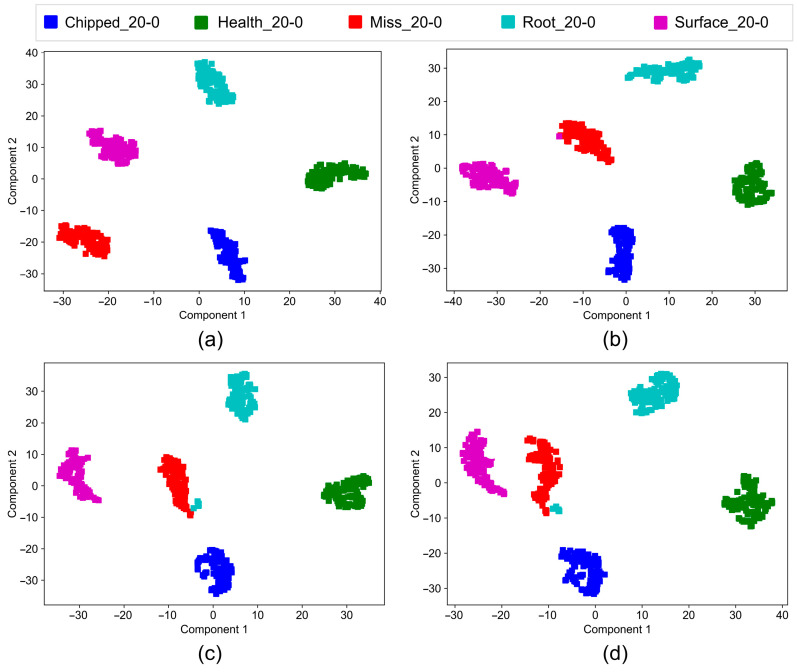
Clustering diagram of CA-DRSN model under working condition 1: (**a**) noise level at 0.1; (**b**) noise level at 0.15; (**c**) noise level at 0.2; (**d**) noise level at 0.25.

**Figure 12:**
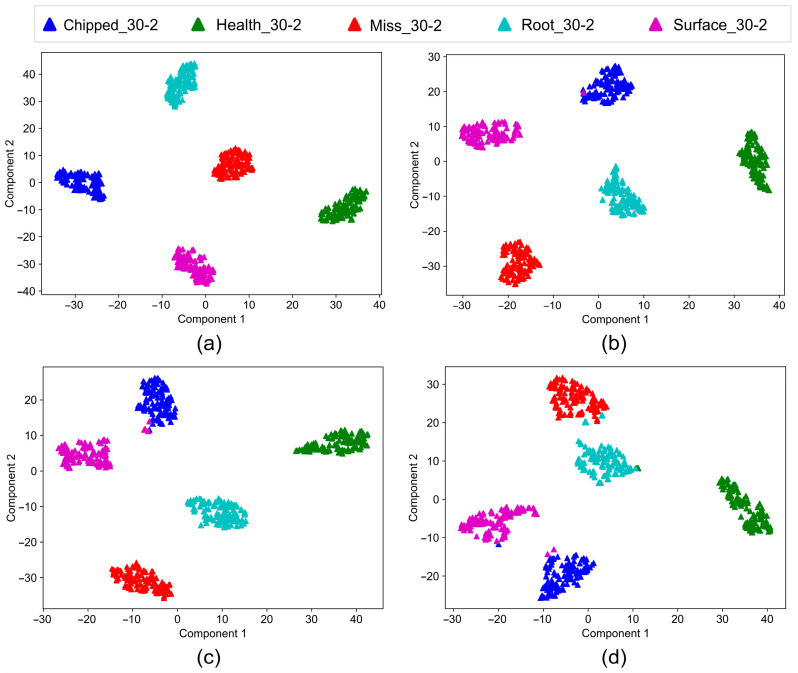
Clustering diagram of CA-DRSN model under working condition 2: (**a**) noise level at 0.1; (**b**) noise level at 0.15; (**c**) noise level at 0.2; (**d**) noise level at 0.25.

The output of the feature vectors is visualized using the t-SNE algorithm for both working conditions, providing a visual representation of the CA-DRSN model’s predictions on the test set. The results shown in Figure 11 and Figure 12 indicate that the division between classes gradually becomes more distinct after training, with five apparent distributions in the connection layer. However, as the noise increases, some data are misclassified, but the overall classification continues to be very effective.

These visualizations further confirm the ability of the model to effectively distinguish between different fault types even in the presence of significant noise, highlighting the robustness and reliability of the CA-DRSN model.

### 4.4. Compound Fault Diagnosis Experiment on PU Dataset

**Figure 13:**
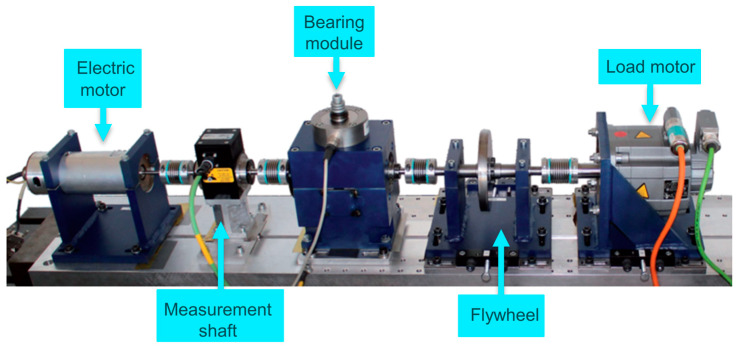
PU experimental device.

Bearings are a critical component of gearboxes. Therefore, experimental data from the Paderborn University (PU) bearing test bench were used to validate the model’s generalization capability. Figure 13 shows the PU bearing test bench, consisting of an electric motor, a torque measuring shaft, a rolling bearing test module, a fly wheel, and a load motor. Bearing fault data were collected at a sampling frequency of 64 kHz, with a shaft speed of 1500 r/min, a radial force of 1000 N, and a load torque of 0.7 Nm.

Since using all data would result in an enormous computational time, only data collected from real damaged bearings were used for performance verification. Each set of 1024 data points was used as a sample, and there were 250 samples in each category. Then, 80% of the samples were randomly selected as the training set, and 20% of the samples were selected as the test set. The specific failure categories and samples for the dataset used in this experiment are shown in Table 4.

As described in Table 5, CA-DRSN maintains the highest average precision at all noise levels, dropping from 0.9766 at the lowest noise level (0.1) to 0.8995 at the highest noise level (0.25). It also performs well in terms of average recall, dropping from 0.9759 to 0.8374. Clearly, the proposed model also exhibits the best accuracy, with the accuracy decreasing from 97.8% to 90.1% as noise levels increase. The relatively low standard deviation indicates stable performance. The performance of DRSN-LSTM significantly declines with increasing noise levels. At the lowest noise level, its precision is 0.9512 and its recall is 0.9463, but at the highest noise level, these values drop to 0.7850 and 0.7836, respectively. The diagnosis accuracy also decreases from 90.9% to 78.8%, indicating that DRSN-LSTM has poor noise resistance. At lower noise levels, DRSN-ECA obtains accuracy and recall values of 0.9649 and 0.9602. These values drop, respectively, to 0.8656 and 0.8192 at the greatest noise level, though. Furthermore, the diagnosis accuracy declines from 96.5% to 88.5%. In contrast, the performance of other models decreases significantly with increasing noise levels, especially AlexNet, which cannot diagnose at high noise levels, indicating low robustness.

In this comparative experiment, CA-DRSN consistently outperforms other models in all evaluation metrics (average precision, average recall, and diagnosis accuracy). It is an ideal choice for real-world fault diagnosis applications because it demonstrates high robustness and reliability even in noisy environments.

## 5. Conclusions

This paper proposes a noise-resistant gearbox fault diagnosis method based on the FFT-transformed CA-DRSN model. Comparative experiments were conducted on SEU gear and PU bearing datasets with different noise levels to validate the effectiveness and robustness of the proposed method. The experimental results show that, compared to existing network models, under low-noise conditions, the proposed model achieved the highest diagnostic accuracies of 99.9%, 99.8%, and 97.8%. In particular, the model has higher accuracy and robustness than other models under high noise. In summary, by means of the incorporation of FFT transformation, cross-attention mechanism introduction, and soft-threshold denoising before and after the first and last residual blocks, the method proposed in this paper significantly enhances the ability to extract fault signals from noisy data. Therefore, local and global information are combined effectively, the characteristic signals for noise and clearly retained faults are reduced, and more accurate and stable fault diagnosis is achieved. The model parameters and experimental results provided also represent valuable data references for existing fault diagnosis research. However, this study only used publicly available datasets, and the actual gearbox operating environment usually contains other types of noise, such as white noise and uniform noise. In future work, these different types of noise should be combined to more accurately simulate actual operating conditions. In addition, more industrial fault datasets from actual work should be combined to verify the composite fault types of weak faults and early faults and therefore further improve the practicality of the model.

## Figures and Tables

**Table 1:** Division of the SEU dataset.

	Type	Health	Chipped	Miss	Root	Surface	Total	Working Conditions
Dataset 1	Training	408	408	408	412	408	2555	20 Hz-0 V
	Validation	102	102	102	103	102		
Dataset 2	Training	408	408	408	412	408	2555	30 Hz-2 V
	Validation	102	102	102	103	102		

**Table 2:** Parameter selection.

Parameter	Value
Epochs	100
Batch size	32
Learning rate	0.001
Learning rate decay	0.1
Optimizer	Adam

**Table 3:** Average precision and average recall of the confusion matrix for seven models under different working conditions.

Model	Noise Level	AP (Condition 1)	AP (Condition 2)	AR (Condition 1)	AR (Condition 2)
CA-DRSN	0.1	1.0	1.0	1.0	1.0
	0.15	0.9942	0.9981	0.9941	0.9980
	0.2	0.9846	0.9868	0.9844	0.9863
	0.25	0.9789	0.9792	0.9785	0.9786
DRSN-LSTM	0.1	0.9976	0.9962	0.9975	0.9961
	0.15	0.9845	0.9818	0.9842	0.9804
	0.2	0.9338	0.9473	0.9256	0.9392
	0.25	0.9324	0.9010	0.9197	0.8628
DRSN-ECA	0.1	0.9974	0.9925	0.9973	0.9922
	0.15	0.9802	0.9797	0.9784	0.9785
	0.2	0.9722	0.9790	0.9716	0.9782
	0.25	0.9472	0.9693	0.9373	0.9682
DRSN-CW	0.1	0.9925	0.9832	0.9922	0.9824
	0.15	0.9847	0.9815	0.9841	0.9804
	0.2	0.9771	0.9781	0.9765	0.9765
	0.25	0.9608	0.9584	0.9589	0.9571
ResNet18	0.1	0.9868	0.9863	0.9962	0.9961
	0.15	0.9606	0.9510	0.9734	0.9727
	0.2	0.9128	0.8804	0.9281	0.9103
	0.25	0.8655	0.8039	0.8851	0.8026
AlexNet	0.1	0.9750	0.9746	0.9885	0.9883
	0.15	0.9721	0.9707	0.9690	0.9669
	0.2	0.9575	0.9550	0.9389	0.9337
	0.25	0.8432	0.8139	0.8787	0.8595
BiLSTM	0.1	0.9730	0.9706	0.9905	0.9902
	0.15	0.9604	0.9589	0.9698	0.9687
	0.2	0.9534	0.9512	0.9375	0.9318
	0.25	0.7045	0.6812	0.8394	0.8068

**Table 4:** Detailed description of the PU university bearing fault dataset.

Bearing Code	Location of Damage	Characteristic of Damage	Extent of Damage	Sample Size
KA04	Outer	single point	1	250
KA15	Outer	single point	1	250
KA16	Outer	single point	2	250
KA22	Outer	single point	1	250
KA30	Outer	distributed	1	250
KB23	Outer and inner	single point	2	250
KB24	Outer and inner	distributed	3	250
KB27	Outer and inner	distributed	1	250
KI14	Inner	single point	1	250
KI16	Inner	single point	3	250
KI17	Inner	single point	1	250
KI18	Inner	single point	2	250
KI21	Inner	single point	1	250

**Table 5:** Confusion matrix analysis and accuracy in fault diagnosis for different models.

Model	Noise Level	Average Precision	Average Recall	Diagnosis Accuracy
CA-DRSN	0.1	0.9766	0.9759	97.8 ± 0.35
	0.15	0.9665	0.9632	96.7 ± 0.52
	0.2	0.9481	0.9454	95.0 ± 0.86
	0.25	0.8995	0.8374	90.1 ± 1.06
DRSN-LSTM	0.1	0.9512	0.9463	90.9 ± 2.20
	0.15	0.8962	0.8740	87.4 ± 1.90
	0.2	0.8804	0.8463	84.6 ± 2.69
	0.25	0.8410	0.7863	78.0 ± 5.80
DRSN-ECA	0.1	0.9649	0.9602	96.5 ± 0.53
	0.15	0.9509	0.9417	93.9 ± 0.93
	0.2	0.9154	0.9094	90.7 ± 1.24
	0.25	0.8656	0.8192	88.5 ± 1.58
DRSN-CW	0.1	0.9354	0.9247	93.3 ± 1.63
	0.15	0.8994	0.8773	89.7 ± 2.20
	0.2	0.8498	0.8235	85.9 ± 2.90
	0.25	0.8105	0.7067	78.9 ± 4.08
ResNet18	0.1	0.9670	0.9602	95.5 ± 0.92
	0.15	0.8985	0.8682	82.0 ± 6.54
	0.2	0.8291	0.7451	72.3 ± 8.26
	0.25	0.7815	0.6515	58.0 ± 6.44
AlexNet	0.1	0.7956	0.7789	77.7 ± 1.61
	0.15	0.7293	0.6870	65.7 ± 1.76
	0.2	-	-	-
	0.25	-	-	-
BiLSTM	0.1	0.9279	0.9265	91.1 ± 0.53
	0.15	0.6107	0.5411	62.7 ± 1.51
	0.2	0.5928	0.5742	57.0 ± 2.15
	0.25	0.5925	0.3856	43.2 ± 2.39

## Data Availability

The dataset used in this article can be obtained from the corresponding author upon request.

## Nomenclature

FFT	Fast Fourier transform
TD Input	Time domain input
AP	Average precision
AR	Average recall
RSBU-CW	Residual Shrinkage Building Unit with Channel-Wise Thresholds
RSBU-CS	Residual Shrinkage Building Unit with Channel-Shared Thresholds
CA-DRSN	Cross-Attention Deep Residual Shrinkage Network
DRSN-LSTM	Deep Residual Shrinkage Network with Long Short-Term Memory
DRSN-ECA	Deep Residual Shrinkage Network with Efficient Channel Attention
DRSN-CW	Deep Residual Shrinkage Networks with Channel-Wise Thresholds
ResNet18	Residual Neural Network 18
AlexNet	Alex Network
BiLSTM	Bidirectional Long Short-Term Memory
